# Developing Key Safety Management Factors for Construction Projects in China: A Resilience Perspective

**DOI:** 10.3390/ijerph17176167

**Published:** 2020-08-25

**Authors:** Zheng Zhu, Jingfeng Yuan, Qiuhu Shao, Lei Zhang, Guangqi Wang, Xuewei Li

**Affiliations:** 1 Department of Construction and Real Estate, School of Civil Engineering, Southeast University, Nanjing 210096, China; 230179806@seu.edu.cn (Z.Z.); jingfeng-yuan@seu.edu.cn (J.Y.); lxwwseu@163.com (X.L.); 2 Mass Transit Divisional Department, CRRC Nanjing Puzhen Co., Ltd., Nanjing 210031, China; 3 China Construction Communications Engineering Group Corp. Ltd., Beijing 100000, China; cscecjtptt@163.com

**Keywords:** construction projects, safety management, resilience, SEM

## Abstract

It is acknowledged that construction safety is pivotal to the project management objectives. Meanwhile, the concept of resilience provides an effective and pragmatic countermeasure to improve the safety management level of construction projects. However, the “resilience” has not gained considerable attention in the construction safety management system. In this context, the paper aims to develop the key safety management factors for construction projects from the resilience perspective. Firstly, the theoretical framework and key safety management factors of construction safety management system based on the resilience theory are proposed. The importance of each factor is then obtained by using the method of structural equation modeling (SEM). The results indicate that information management, material and technology management, organization management and personnel management would improve the safety and resilience of the project. Specifically, improving the resilience of information flow to strengthen the interaction among elements of the system can enhance the safety management level. These findings can be used as references for construction safety managers to improve the abilities of preventing safety accidents and recovering after safety accidents.

## 1. Introduction

For the burgeoning progress in economy and technology in China, the investment on construction projects such as super high-rise buildings, subways and underground complexes are increasing at an astonishing rate. Accompanied by the considerable contributions the construction industry makes, the safety issue in construction projects has become a major concern [1,2]. According to the official report issued by the Ministry of Housing and Urban–Rural Development of People’s Republic of China, 734 accidents and 840 deaths occurred in the construction process of housing and municipal projects nationwide in 2018, up 6.1% and 4.1% from the same period in 2017 respectively. Compared to other labor-intensive industries, the construction industry is more likely to breed safety risks. Due to the fact that the construction project is highly risk prone, the construction safety is of great significance to ensure the achievement of project management objectives [1,3].

For the safety accidents in construction projects, previous studies mainly focused on the dimensions of technology and management [4]. In terms of technology, prior studies attributed the safety issues to the construction method, soil displacement, mechanical performance and other technical design issues [5]. For example, Leu and Chang [6] assessed the steel construction safety risk by Bayesian networks based on fault tree. Likewise, the management dimensions laid emphasis on analyzing dangerous events, hazard sources and safety accident occurrence mechanism, so as to fulfill the purpose of monitoring, analysis and early warning of the safety accidents [7,8]. For example, Nahangi, Chen and McCabe [8] developed a safety performance framework for the construction projects and identified the factors exerting the greatest impact on safety performance based on the data envelopment analysis (DEA) method. In terms of these two dimensions, the application of advanced technology has gained more attention, but the management dimension is in urgent need of improvement [9,10]. Therefore, this paper has screened the management dimension as the objective of optimization. In the management dimension, the administration of the construction project is complex, as there still exists significant problems in the safety domain [11]. For example, problems such as unclear organization interface and unimpeded information flow, have restricted the efficiency of safety management of construction projects. As the case may be, it is of paramount significance to adopt scientific and effective approaches to improve the management level of safety in the construction projects [12].

The concept of resilience has provided a measure to tackle the managerial complexities in construction projects [13,14]. It depicts the ability of “defense” before system damage and “recovery” after system damage, and has been gradually introduced in safety management in construction projects [15,16]. What should be noted is that “resilience” does not occur naturally. Instead, the resilience is better to be designed into the system in order for the system to possess some type of resilient behaviors [14,17]. In construction projects, managers are advised to redesign the project organization, safety management process and other management elements to ensure the concept of resilience dose play a more crucial role in the construction projects. However, contemporarily, the application of resilience in the safety management of construction has not been paid enough attention to, and there are few studies probing the factors, which potentially affect construction safety management in the context of resilience and the importance of these factors [17].

Therefore, this paper aims to develop the key safety management factors for construction projects in China from the resilience perspective. In order to provide a theoretical foundation for the key safety management factors, a theoretical framework of construction safety management system based on the resilience theory are firstly defined according to the prior studies. Further, the key factors affecting construction safety management in the whole process of accident occurrence are identified from four dimensions, namely organization management, personnel management, material and technology administration and information management. Thereafter, the importance of each factor is obtained by using the method of structural equation modeling (SEM).

## 2. Literature Review

### 2.1. The Concept of Resilience

The term, resilience, has been frequently seen in the research literature. Haimes [18] defined the resilience as “the ability of a system to withstand a major disruption within acceptable degradation parameters and to recover with a suitable time and reasonable costs and risks”. Hosseini, Barker and Ramirez-Marquez [14] indicated that the common use of “resilience” implies the ability of an entity or a system to return to the normal condition after the occurrence of an event that disrupts its state. As an inherent ability of the system, resilience has many definitions, but its essence emphasizes the system’s ability to absorb, adapt, recover and improve external interference (such as accident), and has the attributes of situational awareness, resistance, robustness, adaptability, recovery, timeliness, learning ability, etc. [16].

The concept of resilience adopted in a system can be considered from two aspects, namely the accident response process and management elements [19]. The accident response is an accident process-orientated perspective, which focuses on the operation process, having the properties of workflow [16,20]. With respect to the accident response, there are two main perspectives to define the resilience. The first one is in terms of defense and control, whose purpose is to mainly deal with accidents, and can be named “defense resilience” [19]. The second one is to consider in terms of recovery and adaptation, whose aim is to adjust strategies for accident change, and can be named “recovery resilience” [19]. In the construction projects, these two perspectives generally aim at different stages of project management. Defense can be understood as prevention in advance and control in the process, which is to minimize the impact of accidents on the project. Recovery can be interpreted as learning and adjustment after the event, reflecting on the measures in advance and in the event, and adjusting the structure to improve its impact resistance.

The management elements are the foundation of the system with defense resilience and recovery resilience. The aspect of management elements in the resilient system mainly focuses on the relationship between organization operation and external constraints [19]. It is argued that the external constraints dictate the structures and the internal life of an organization. The resilience-oriented organization can efficiently perceive changes in its environment and respond appropriately to them [19]. Hosseini, Barker and Ramirez-Marquez [14] indicate that the resilience of an organization is the inherent ability to keep or recover a steady state, thereby allowing it to continue normal operations after a disruptive event. The aspect of management elements mainly prevents accidents through considerate organizational design and management. For example, organizations need to decentralize and empower front-line employees to report and deal with accidents quickly. Meanwhile, “culture of reliability” has an important role in organization, which will affect the behaviors of employees [19].

### 2.2. Application of Resilience in Construction Projects Safety Management

The poor safety management increases the possibility of construction project failures and influences all other key performance indicators [21,22]. For example, unsafe work environment will undermine the quality of work and consequently incur additional time and subsequent costs due to such conditions [23]. Therefore, safety management has attracted the concern of construction project managers [19]. However, there are still problems demanding prompt solutions in the construction safety management process [24]. For example, some safety management activities are reactive, defensive and fragmented, with deficiencies of active exploration and learning, failing to pay enough attention to the prevention ability and slacking on systematic planning. Since the concept of resilience was coined during the Söderköpinge meeting, it has received an increasing amount of attention in the safety management system. Resilience emphasizes the defense, recovery, learning and other abilities of the system, and provides a new way for improving the safety management [16,25,26,27].

In the aspect of an accident response, the resilience in construction projects safety management is related to the ability of project systems to perceive risks, predict accidents, take emergency measures and learn accident experiences [13,16,25]. In this term, the safety resilience is the ability of the system to maintain, restore and optimize the safety state of the system when facing the disturbance of safety risks within a certain time and space [16].

Regarding the management elements, factors affecting construction projects safety resilience include organization, technology, information and process, external economic conditions, employees’ psychological factors, etc. [7,11,28,29]. The organization refers to the top management policies and administrative safety activities, which is the foundation of safety practice [30]. The technology refers to any practice that ensures a safe working environment, including safety planning [31]. The employee’s psychology refers to any practices changing human knowledge and cognition, considering safety practices directly affecting the worker [11]. The information refers to the flow of information between and within organizations in the safety management process of construction projects.

Many existent studies have focused on the resilience in construction projects safety management and proposed the safety management factors. For example, Peñaloza, Saurin and Formoso [20] used the resilience assessment grid (RAG) to monitor the construction projects safety management. Guo, Amin, Hao and Haas [32] developed the assessment index system for safety resilience of the subway construction site. Ranasinghe, Jefferies, Davis and Pillay [17] reviewed the literatures exploring resilience engineering (RE) indicators that had been used to assess the level of resilience in high safety risk industries, particularly in construction projects. The safety management factors proposed in these existing studies were mainly identified from the perspective of system management elements. The whole process of the safety accident response and construction project life cycle has not been fully considered in these studies. However, the perspective of the safety accident response process is of paramount significance for strengthening the coordination of various factors in a life cycle and improving the management level systematically [33,34]. Therefore, this paper has not only taken the management elements into account, but also considered the safety accident response process when developing the safety management factors.

### 2.3. Theoretical Framework and Hypotheses

**Figure 1:**
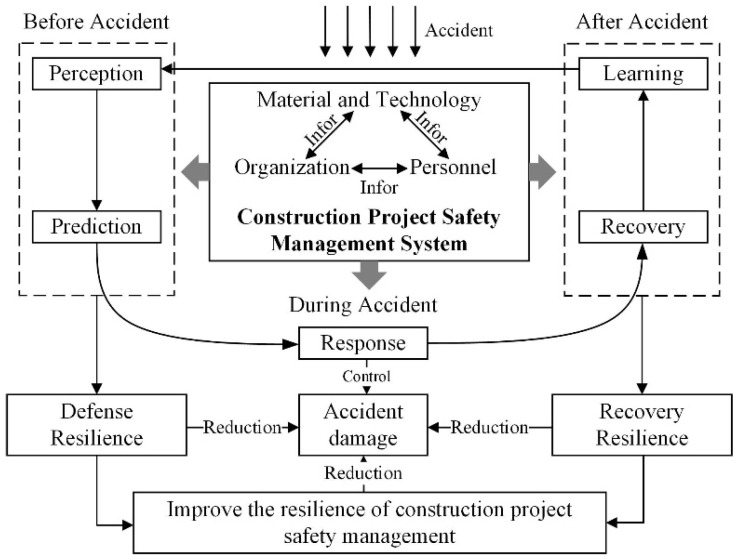
The theoretical framework of construction safety management based on resilience theory.

Based on the above review of resilience and safety management [12,16,20,23,35,36,37,38], this paper defines the safety resilience in construction projects as the ability of perception, prediction, response, recovery and learning of complex construction systems composed of organization, personnel, material, technology and information under the disturbance of external environment in each stage before, during and after an accident. A theoretical framework is designed as shown in Figure 1, which is developed from two aspects, namely safety accidents response process and the management elements.

In the theoretical framework, the safety resilience of the construction system primarily includes two parts from the perspective of accident response process: accident defense resilience and recovery resilience [33,34]. The accident defense resilience mainly refers to the ability of the safety management system to prevent and control safety accidents. The accident recovery resilience mainly refers to the recovery efficiency of the safety management system due to safety accidents. The accident defense resilience and recovery resilience can be further divided into five dimensions: perception ability, prediction ability, response ability, recovery ability and learning ability. Before the accident, “resilience” requires the safety management system to be able to feel the accident and predict the impact of the accident. When the accident occurs, the “resilience” requires that the safety management system is capable of controlling the accident well and minimizing the wastage caused by the accident. After the accident, resilience requires the safety management system to have a better emergency and rescue system, and to be able to recover as quickly as possible from the accident attack. Meanwhile, after the accident, the “resilience” also requires the safety management system to effectively summarize the safety management process and learn from the experience.

According to the management elements of the construction safety management system and the information transmission among the elements, the basic elements of construction safety resilience contain organization, personnel, material, technology and information. In the construction project, the four elements are coupled with each other and eventually construct the safety management system. Therefore, this paper aims to enhance the resilience of the safety management system from these four elements, so that the system can obtain defense resilience and recovery resilience.

The organizational management is related to the organizational model, organizational structure, management system, etc. In construction projects, safety management and organizational elements constitute the principles of conduct for safety management system through formal system, determining the safety awareness of managers and employees through informal system (such as organization culture) [37,39]. Meanwhile, Xie and Guo [40] indicated that poor implementation of the institution, inadequate supervision and failure to correct a known problem and supervisory violations in the aspect of organization will influence the resilience of the safety management system. Therefore, organization is one of the key avenues to improving safety resilience. The following hypotheses can be introduced:

**Hypothesis** **1** **(H1).** *There is a positive relationship between the organizational management and the safety management resilience of construction projects*.

The personnel management includes two aspects: managers and workers. The resilience of managers tend to improve the organization and personnel management ability, while the safety resilience of workers tends to improve the safety production skills and attitudes of workers [36,41]. Saurin, Formoso and Cambraia [38] indicated that the employee’s abilities, skills and psychology will have an impact on the efficiency of the accident response before the accident and the knowledge acquisition after the accident in the safety management system, which are significant for safety resilience. Therefore, the following hypotheses can be introduced:

**Hypothesis** **2** **(H2).** *There is a positive relationship between the personnel management and the safety management resilience of construction projects*.

The material and technology management not only refers to the management of material resources and mechanical equipment used in the process of construction, but also the equipment or technical innovation that carry weight on the construction safety [42]. The material and technology management influence the “safe state of material and equipment” and the “safe behavior of people”, which are the basic elements for the normal exertion of the resilience function of the safety management system [20,43,44]. Therefore, the following hypotheses can be introduced:

**Hypothesis** **3** **(H3).** *There is a positive relationship between the material and technology administration and the safety resilience of construction projects*.

Information acts as the “communication” bridge between managers, environment and system elements in the construction safety management system, and it is the enzyme medium and dynamic mechanism of the construction safety management and function control model, which makes up for the lack of consideration of information transmission resilience among system elements in the traditional safety management system. Wehbe, Al Hattab and Hamzeh [23] pointed out that construction project organizations with timely and tightly connected interactions have higher information transfer density, so as to have higher resilience to risk prediction ability and better safety performance. Qian and Lin [12] indicated that organizations with efficient information transfer and rapid feedback will have a higher safety accident response ability, so as to have a better resilience ability. Therefore, the following hypotheses can be introduced:

**Hypothesis** **4** **(H4).** *There is a positive relationship between the information management and the safety management resilience of construction projects*.

**Figure 2:**
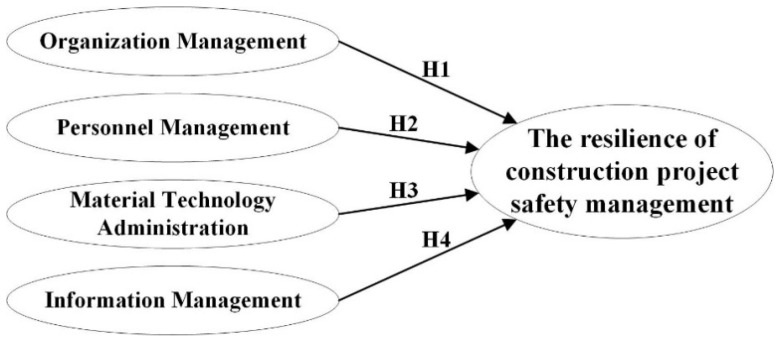
The hypothetical model.

Based on the above hypothesis, the hypothetical model is as shown in Figure 2.

## 3. Research Method

The research phases developed for this paper are as follows.

Firstly, according to the theoretical framework, this paper developed a preliminary list of construction safety management factors based on the resilience theory. In this preliminary list, the construction safety management factors mainly refer to the management elements in the theoretical framework, and correspondingly, the related resilience types of each factor are also identified, which mainly refers to the safety accidents response process in the theoretical framework.

The preliminary factors are identified based on the existing studies. However, some factors from existing studies may be a similar, redundant or inaccurate representation [45,46]. Therefore, in order to delete and adjust preliminary factors, a pilot questionnaire survey was then conducted to screen the factors based on the exploratory factor analysis (EFA) model, and then the formal questionnaire was designed based on the results of EFA.

Based on the results of EFA, the formal questionnaire survey was conducted, aiming to collect the judgments provided by a group of experts regarding the relationship between factors. The structural equation modeling (SEM) was then used to measure and quantify these relationships between factors. The key factors can be identified based on the results of SEM.

Finally, the characteristics, contributions and practical implications of the model and factors of construction safety management based on resilience theory would be further discussed.

**Figure 3:**
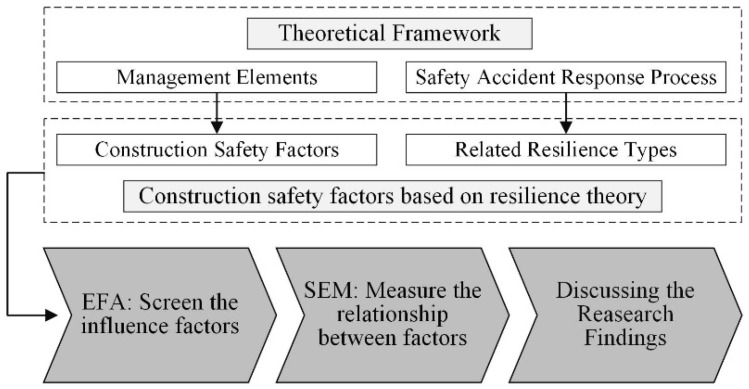
The research phases developed for this paper.

The research phases are developed as shown in Figure 3.

### 3.1. Exploratory Factor Analysis (EFA) and Structural Equation Modeling (SEM)

In multivariate statistics, EFA is a statistical method used to uncover the underlying structure of a relatively large set of observation variables (usually indicators or factors). EFA is a technique within factor analysis whose overarching goal is to identify the underlying structure between observation variables, so as to realize the classification or deletion of observation variables [45,46]. This technique can be applied to identify data structures in questionnaires, streamline the data where appropriate, and reduce the variables in the questionnaire into smaller numbers [11,47]. The EFA has been used in some existing studies to simplify the factors. For example, Zaira and Hadikusumo [11] used the EFA to simplify the safety intervention practices affecting the safety behavior of workers in the construction industry. Liu, Zhao, Zhou and Tang [47] employed the EFA to simplify the safety risk factors of metro tunnel construction.

However, since there is no factor model that is a perfect representation of reality, the correlation problem and other problems will still exist in the factors simplified by EFA [11,45,47]. Therefore, this paper used the SEM to identify the key safety management factors, so as to further refine information. SEM is a statistical data analysis tool that synthetically uses multiple regression analysis, path analysis and factor analysis [46]. SEM is aimed to explore questionnaires or other empirical data, which is essentially a hypothesis testing model analysis or confirmatory factor analysis. It attempts to use the empirical data collected by researchers to establish, evaluate and test the relationship between one or more independent variables and one or more dependent variables, so as to identify the importance of different variables [46,48,49,50].

### 3.2. Data Collection

To explore the construction safety factors based on resilience theory, a top-down approach was adopted. This approach indicates that the set of indicators were developed based on the proposed theoretical framework as shown in Figure 1 and some prior studies [51]. According to the theoretical framework, the prior studies of resilience and safety management [12,16,20,23,24,25,26,30,31,35,36,37,38,40], four dimensions that constitute safety management objects in construction projects are proposed, and thirty construction safety management factors based on resilience theory are screened out, as shown in Table 1. Meanwhile, different factors will affect the types of system resilience at a different level (including defensive resilience and recovery resilience), and the related resilience types of each factor are also contained in Table 1.

A questionnaire survey was designed to investigate the positive impacts of the construction safety factors based on the resilience theory. The targets of this survey are professionals and informed experts, who have local and international experiences in construction safety management and resilience. The questionnaire was divided into two parts, and each question was built as a single-choice in a structured format. The contents of the questionnaire are shown in Table 2.

The questionnaire survey was structured to spend approximately 10–15 min to complete, which was conducted in September 2019 with two steps. The first step was a pilot survey. A total of 180 questionnaires were collected through an online approach in a pilot survey, and 161 valid questionnaires were finally retained. The results of a pilot survey were then analyzed by EFA, which aims to screen the influence factors. The second step was to amend the questionnaire according to the pilot survey and conduct the formal questionnaire survey. Some respondents were selected for the formal survey from the respondents in the pilot survey, a total of 151 questionnaires were collected through an online approach in a formal survey, and 139 valid questionnaires were finally retained. The collected data was analyzed by SEM, aiming to identify the key safety management factors.

## 4. Data Analysis

### 4.1. Demographic Characteristics

According to 161 valid questionnaires in a pilot survey, the age, educational background, working years and positions of all the subjects were statistically analyzed. The characteristics of the sample data are shown in Table 3. Through the analysis of sample distribution with the same population attribute, it can be seen that the data obtained from this questionnaire survey was complete, uniform and reasonable, so that the credibility of the conclusions of the model could be guaranteed.

### 4.2. Exploratory Factor Analysis

Using SPSS 25.0 (a statistical software developed by IBM in Armonk, NY, USA), the initial reliability and exploratory factor analysis were carried out on the scale items in the questionnaire, so as to adjust the preset dimensions, items of the scale and eventually form a formal questionnaire. Firstly, the initial reliability of the sample data was analyzed. The corresponding Cronbach’s alpha α (α) of total dimensions and factors was 0.966, the α of the organization management dimension and factors was 0.842, the α of the personnel management dimension and factors was 0.898, the α of the material and technology administration dimension and factors was 0.924, the α of the information management dimension and factors was 0.853. All the α were greater than 0.80, indicating that the reliability of the scale was good [46]. Simultaneously, according to the reliability coefficient after deleting items, the two factors of “large-scale machinery management” and “temporary facilities management” were eliminated. Therefore, there were 28 factors that remained as shown. Secondly, Kaiser–Meyer–Olkin (KMO) and Bartlett spherical test were performed to identify the correlation between individuals. On a basis of the data in Table 4, KMO is 0.935 (greater than 0.9), Bartlett spherical test *p* = 0.000 (less than 0.01), revealing that the sample data are highly relevant and suitable for factor analysis [46].

In the exploratory factor analysis, this paper adopted the method of the principal component analysis. This paper set the factor extraction number to 4 according to the preset dimension, and characteristic roots of the four dimensions were 16.006, 3.625, 1.317 and 1.031 respectively, which were all greater than 1 and had the 68.688% explanatory power. Therefore, the optimal skew method was used to rotate, and the mode matrix of factors of construction safety management was obtained, as shown in Table 5. According to the pattern matrix, the influence factors could be divided into four dimensions, which were consistent with the preset dimensions.

Based on the results of exploratory factor analysis, the content of the questionnaire was adjusted to build a formal questionnaire. According to Xue et al., the following factors will be deleted: (1) factors with inaccurate representation and (2) factors with low factor loading values [46].

The dimensions of each factor obtained through the exploratory factor analysis were compared with the preset dimensions, and there were conflicts in six factors: workers’ education level, workers’ experience and skills, workers’ receiving safety training, investment in safety production expenses, accident emergency response system and post-disaster recovery system. However, the meanings of these six factors were significantly different from the dimensions they belong to, which were obtained through the exploratory factor analysis. The workers’ education level, workers’ experience and skills and workers’ receiving safety training were related to the personal dimensions but not the material and technical dimensions, the investment in safety production expenses was related to the material and technical dimensions but not the information dimensions, the accident emergency response system and post-disaster recovery system was related to the organization dimensions but not the personal dimensions.

Meanwhile, these six factors were broadly similar to some factors and could be expressed by the other factors. The workers’ education level, workers’ experience and skills and workers’ receiving safety training was related to the completeness of organizational structure, workers’ safety attitude and other factors. The investment on safety production expenses was related to the safety guarantee materials on the construction site. The accident emergency response system and post-disaster recovery system was related to the completeness of the organizational structure. Therefore, these six factors could be deleted to simplify the safety management factors.

Furthermore, the dimension of material and technical management had more impact factors. In order to highlight the key factors, the two factors with low factor loading values in the material and technology management dimension were deleted, which were dangerous material management and workers’ safety protection equipment and living conditions guarantee. These two factors were similar to each other and could be expressed by the factor of safety guarantee materials on the construction site. Therefore, these two factors could be deleted to simplify the safety management factors. Finally, 20 key influence factors were obtained, as shown in Table 6.

### 4.3. Confirmatory Factor Analysis

**Figure 4:**
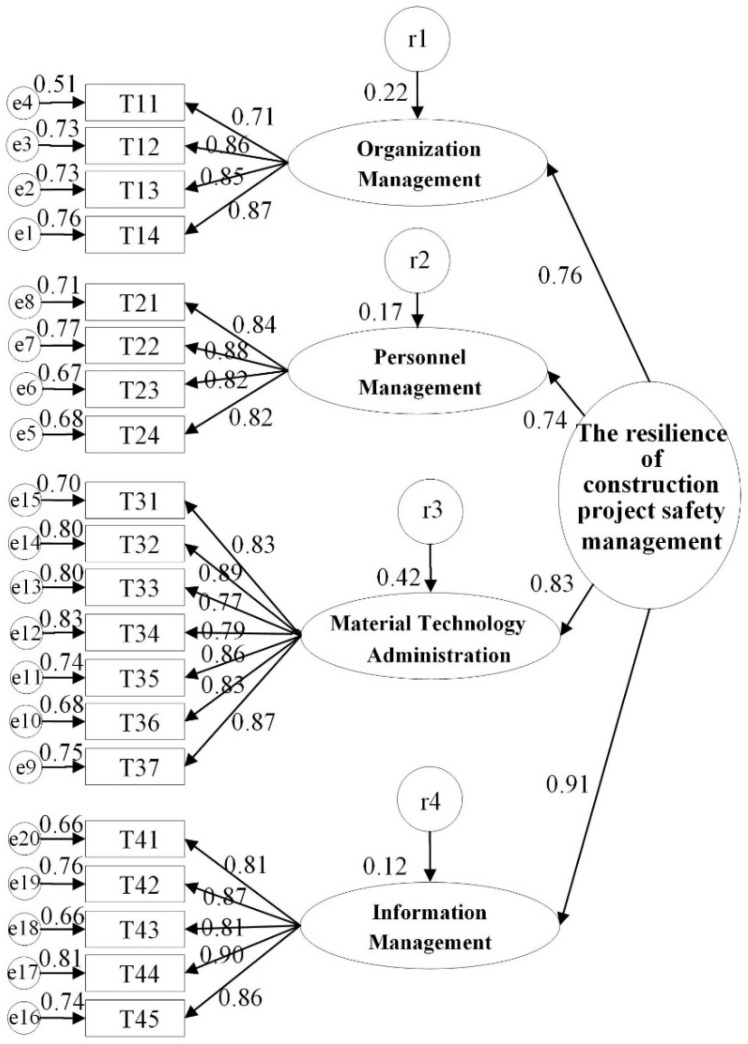
Road map for the SEM model.

A road map for the SEM model related to the influence factors of construction safety management were firstly developed, which reflected the hypotheses of four dimensions with the safety management resilience of the construction project. A total of 139 valid questionnaires were collected from the formal questionnaire survey. Based on the questionnaire data, AMOS 24.0 (a statistical software developed by IBM in Armonk, NY, USA) was used for the SEM to verify the validity and fit of the four-dimensional model of the organization-personnel-material technology-information. Maximum likelihood estimation was used for model parameter estimation, and the standardized solution after data fitting is shown in Figure 4.

The fit index of model is shown in Table 7. The absolute fit index CMIN (likelihood-ratio chi square)/df (degree of freedom) = 1.732 < 5, and the absolute fit index RMSEA (root mean square error of approximation) = 0.073 < 0.08. The other absolute fit indexes CFI (comparative fit index) and TLI (Tacker-Lewis index) were both higher than 0.9. Therefore, the fit index of the model meets the requirements. With confirmatory factor analysis, it can be seen that the model had a well fit index for interpreting the relationships between the impact factors and the resilience of construction project safety management. Therefore, it is unnecessary to take further modification.

The C.R. (critical ratio) and *p* value were utilized to test the statistical significance of factor loading values. If all *p* values are lower than 0.05, which indicates that the road map coefficients are significantly different with zero at the confidence level of 95%. The testing results are shown in Table 8 and all the *p* values were lower than 0.05. It can be concluded that almost all relationships between the four dimensional of organization-personnel-material technology-information and the resilience of construction safety management were significant at the 95% confidence level. Therefore, the four hypotheses in this paper can be verified.

## 5. Discussion

### 5.1. Improving the Resilience of Information Flow to Strengthen the Element Interaction of the System

**Figure 5:**
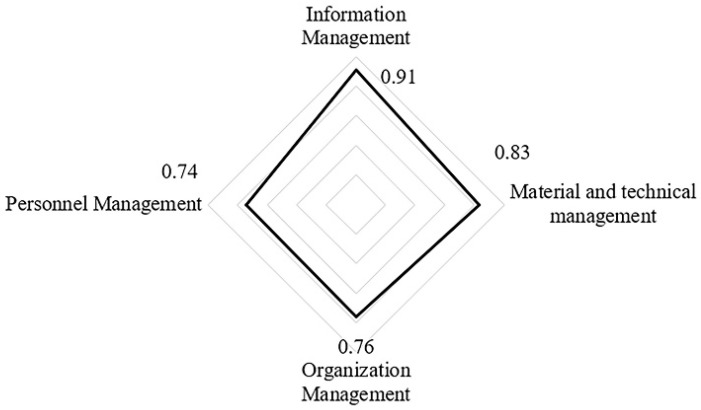
Validity of the four dimensions.

Through EFA and SEM, this paper verified that effective organization management, personnel management, material and technology management and information management could improve construction safety and resilience in the whole process of accidents. According to the priority of each factor in SEM model (as shown in Figure 5), it can be concluded that information management was the most significant factor affecting construction safety and resilience in the management process, followed by material and technology management, organization management and personnel management.

More specifically, for the information management, improving the effectiveness and efficiency of risk identification and analysis and safety information transmission, and strengthening the accuracy of safety information transmission were the key factors (T44 and T42, the load factors were 0.90 and 0.87 respectively). The construction safety management is a process of multi-elements cooperation [52]. Under the theoretical framework of safety resilience, the continuous interaction of organization, personnel and other system elements can promote the improvement of system resilience. Improving the resilience of information flow generated by element interaction is a crucial path for improving the system resilience [53]. Safety professionals established intelligence-gathering lines of communication to key people and data systems across the organization. This intelligence includes: people changes, resources scarcity, operational shifts, goal conflict or changes in the external operating context of the organization. This real-time information provides the safety professional with insight for where safety risk may be increasing, trade-offs occurring and safety margins eroding [54]. Therefore, improving the effectiveness, efficiency and accuracy of information transmission are of great concerns and significance.

In the aspect of material and technology management, improving the handling efficiency of risks and safety accidents, strengthening emergency drills and promoting safe and civilized construction were the key safety management factors (T32, T37 and T35, the load factors were 0.89, 0.87 and 0.86). The construction equipment is considered to be one of the weakest links in the construction industry: site operations are still rather primitive due to the shortage of practical hand tools [29,53]. The abundant supply of cheap labor further exacerbates the situation, causing the construction industry to lag behind in technology [55]. In order to lower the high risk of technology and equipment, strengthening emergency drills and promoting safe and civilized construction is of great necessity [56].

For the management of organizations and personnel, the focus is to formulate a consummate risk management system and a safety supervision system, establish a complete and mature organizational structure, including safety management organization, emergency management organization, post-disaster recovery organization, etc. (T14 and T12, the load factors were 0.87 and 0.73), and prompt the organization and emergency response ability of personnel management and the safety attitude of workers (T22 and T21, the load factors were 0.88 and 0.84). An impeccable risk management system can effectively improve the emergency response capacity of construction projects and improve the defense resilience. The supervision mechanism can guarantee the effective implementation and the quality of various management measures. As the specific executor of safety management activities, the safety attitude and safety management ability of workers directly influence the safety management level of construction projects.

### 5.2. The Key Safety Management Factors

Table 9 selected the key safety management factors based on the factor load in the SEM model. Meanwhile, the factors in Table 9 are distinguished into affecting defense resilience factors (before event) and affecting recovery resilience factors (when/after event) according to the types of impact on the construction safety of each factor in Table 9.

Existing factors related to the resilience and construction safety management are mainly identified from the perspective of system management elements [16,17,22,28]. However, the construction safety management should also pay attention to the whole process of accidents [24]. Therefore, compared to these existing factors, the key safety management factors proposed in this paper not only consider the management elements, but also consider the related resilient types (defense resilience or recovery resilience). The related resilient types of each factor are from the perspective of the safety accident response process, which is helpful to strengthen the coordination of various factors in the life cycle and improve the management level systematically [33,34]. In this context, from the perspective of an accident response process in the life cycle of the safety resilience system, the paper further analyzed the impact of various factors on the resilience ability, which includes perception ability, prediction ability, response ability, recovery ability and learning ability.

Before the accident, as the criterion for the integration of elements in the whole safety resilience system, a clear set of mission and goals must be set through the establishment of a complete organization and the formulation of a perfect management system [57]. When the safety management goal is clear, the safety resilience system will have a clear perception of security accidents.

Thereafter, strengthening the effectiveness and efficiency of safety risk management and other information transmission are the key factors to improve the prediction ability of the safety resilience system [58]. Before the accident, risk prediction is often more important than perception ability [58]. However, construction project managers have difficulty identifying hazards [59,60]. This may be due to the lack of real and timely information. As the safety management information is the main input for risk prediction, the effectiveness and efficiency of its transmission are significant to the identification of hazards.

When the accident occurs, the manager is advised to improve the handling efficiency of safety accidents through emphasizing the role of managers. In this dynamic work environment, the role of the construction manager, who is directly in charge of construction operations and their management on a daily basis, is crucial [61]. The role of the foreman or the superintendent has been identified, both by managers and workers, as one of the most powerful influences on the safe work behaviors of workers [62]. Perlman, Sacks and Barak [58] concluded that operatives see their superintendent’s attitude towards safety as a major source of influence upon their behavior on site. Therefore, improving the organizational and emergency response ability of managers is pivotal. More importantly, the supervision can enhance safety behavior [63].

After the accidents, the recovery ability and learning ability should be given priority by the safety management system. In this aspect, the human error is one of the good reasons for construction industry accidents [64,65,66]. Therefore, it has been noted that the occupational training for the workers plays a vital role in safety at work in the construction industry [67,68]. Tam, Zeng and Deng [69] indicated that safety training is a main safety practice in reducing accident rates, the purpose of which is to provide the compulsory knowledge related to safety that should be known by workers to guide them in how to work in a safe manner [70,71,72]. The consensus is that the safety training can improve the safety awareness and competency of the workers.

### 5.3. Practical Implications and Recommendations

As a diversity of innovative technologies continue to develop, the application of cutting-edge technologies can be an effective solution to safety issues of construction management [73,74]. Baek and Choi [42] indicated that in terms of physical support, the construction projects should introduce information-based equipment and facilities to improve the quality and efficiency of information management in the safety management system. Through big data, artificial intelligence and other technologies, the information resources of project management and safety management can be planned, organized and controlled systematically by the managers. Therefore, the turnover efficiency and response speed of information in the safety management process can be further optimized, and the purpose of improving the toughness of information transmission and promoting the interaction of various elements of the organization and personnel can be achieved.

In the construction projects, the reluctance to input resources for safety is a vital factor for safety management [75]. This is closely associated with the operational nature of construction firms in China. With the rapid urbanization, while the number and size of Chinese construction enterprises have experienced a skyrocketing growth, China’s construction market is highly competitive, which results in excessive competition and thin profit margins. Therefore, subject to the cost, most enterprises are reluctant to carry out substantial organizational reform [68]. In this point, Demirkesen and Arditi [36] and Chen, McCabe and Hyatt [76] indicated that an appropriate reward and punishment system, safety culture training of employees and other less cost soft measures have strong applicability, which can achieve a balance between the administration cost and improvement of safety management. For example, safety incentive is one of the proactive techniques utilized by management to motivate employees to work safely, which can be financial or nonfinancial awards [29]. Meanwhile, in the human–computer interaction interface and other elements of the information management physical support system, the manager can set humanized warning signs and fragmented safety knowledge training, and establish a safety culture atmosphere to eliminate unsafe physiological and psychological factors such as staff fatigue and inertia.

## 6. Conclusions

The new research field of resilient construction provides a promising path to improving the construction safety management since resilience can provide mega-projects with means and technologies to withstand daily problems, unexpected disruptions and even unexampled events as less harm as possible. However, current construction safety management pays less attention to the theory and practice of resilience. Therefore, this paper introduces the concept of resilience in construction safety management to build a theoretical framework of construction safety management on a basis of resilience theory. This theoretical framework is related to the accident defense resilience and recovery resilience, and contains the elements of organization, personnel, material, technology and information. Based on the theoretical framework, this paper then identifies the impact factors of project safety management. Furthermore, this paper obtains the relationship and importance of factors through EFA and SEM. The conclusions are as follows:

(1) The safety resilience of the project is determined by both the accident defense resilience and the recovery resilience. The defense capability of the project should be optimized before the accident, and the recovery capability of the project should be enhanced when the accident occurs and after the accident, so as to realize the goal of the safety resilience of the project by the optimal means.
(2) In the life cycle of accident development, through information management, material and technology management, organization management and personnel management, the safety and resilience of the project will be comprehensively improved. According to the results of SEM, although the four dimensions differ in priority, they have little difference in importance (in order is 0.91, 0.83, 0.76 and 0.74), so they should all be paid great attention to in the construction safety management.
(3) Comparatively speaking, the information management has the greatest influence on the safety and resilience of the construction project. This factor is an intermediary to enhance the interaction of various elements in the management system and to thoroughly promote system resilience. Therefore, the level of information management in construction projects should be consistently improved by means of information technology in the future.

It should be noted that although information is a relatively notable factor, compared with organization, technology and other elements, it is subject to less institutional constraints, or is more likely to be tampered with. The tampered information cannot reflect the real situation, which provides conditions for managers and employees to take opportunistic behaviors by using information asymmetry and information fraud, so as to weaken the resistance ability of the security management system from the inside [12,23]. Therefore, in future research, how to ensure the authenticity of information and how to establish information supervision mechanism are worthy to study. The recommendations related to the information resilience proposed in this paper, such as organizing and controlling the information resources of project management and safety management, can be refined into management models or specific measures through empirical research in the future.

## Figures and Tables

**Table 1:** Construction safety factors based on resilience theory.

Dimension	Source	Influence Factors	Related Types of Resilience
Organization Management	[12,16,20,25,30,35,37]	Rationality of organizational model	Defense resilience Recovery resilience
		Completeness of organizational structure	Defense resilience Recovery resilience
		Safety supervision system	Defense resilience
		Risk management system	Defense resilience
		Accident Emergency Response System	Recovery resilience
		Post-disaster recovery system	Recovery resilience
Personnel Management	[16,20,23,24,30,35,36,40]	Safety accomplishment of managers	Defense resilience
		Organizational competencies of managers	Defense resilience Recovery resilience
		Emergency response capability of managers	Recovery resilience
		Workers’ safety attitude	Defense resilience
		Workers receive safety training	Defense resilience
		Experience and skills of workers	Defense resilience
		Education level of workers	Defense resilience
Material and Technology Administration	[24,26,30,31,36,38]	Technical disclosure	Defense resilience
		Civilized construction	Defense resilience
		Emergency drills	Recovery resilience
		Risk control	Defense resilience
		Handling of safety accidents	Recovery resilience
		Investment in safety production expenses	Defense resilience Recovery resilience
		Workers’ safety protection equipment and living conditions guarantee	Defense resilience
		Safety guarantee materials on construction site	Defense resilience
		Large-scale machinery management	Defense resilience
		Dangerous material management	Defense resilience
		Temporary facilities management	Defense resilience
		Emergency equipment management	Recovery resilience
Information Management	[12,20,25,30,35,36,37]	Hazard source monitoring	Defense resilience
		Identification and analysis of risks	Defense resilience
		Sharing and learning of safety knowledge	Defense resilience Recovery resilience
		Efficiency of security information transmission	Defense resilience Recovery resilience
		Accuracy of safety information transmission	Defense resilience Recovery resilience

**Table 2:** The content of the questionnaire.

Part	Questionnaire Content
1	The background information of respondents, including 4 questions of ages, level of education, working years and position.
2	Evaluating the importance of each factor that may affect the safety and resilience of the construction project. The Likert’s 7-level scale is used to indicate the attendees’ view on the listed question in the part 2, 1 = very unimportant, and 7 = very important.

**Table 3:** Demographic characteristics of respondents (*N* = 161).

Attribute		*n*	Percentage
**Age**	20–30	71	44.1%
	31–40	66	41.0%
	41–50	17	10.6%
	More than 50	7	4.3%
**Level of education**	Postgraduate or above	47	29.2%
	Undergraduate	49	30.4%
	Junior college	18	11.2%
	High school, technical secondary school or vocational high school	26	16.1%
	Other	21	13.0%
**Working years**	More than 10 years	42	26.1%
	6–10 years	40	24.8%
	1–5 years	44	27.3%
	Less than 1 year	35	21.7%
**Position**	Company management personnel	21	13.0%
	Project management personnel	38	23.6%
	Ordinary employees	63	39.1%
	Construction personnel	14	8.7%
	University researchers	25	15.5%

**Table 4:** Kaiser–Meyer–Olkin (KMO) and Bartlett spherical inspection.

Inspection Items		Inspection Value
KMO		0.935
Bartlett spherical test	Approximate chi-square	4561.016
	df	496
	sig.	0.000

Note: df is the degree of freedom, sig. is the significance value.

**Table 5:** Model matrix and dimension comparison of influence factors of construction safety management.

Influence Factors	Dimension	Composition				Default Dimension
		1	2	3	4	
Risk control	Material and technical management	0.985				Material and technical management
Education level of workers		0.965				Personnel Management
Handling of safety accidents		0.815				Material and technical management
Emergency equipment management		0.803				Material and technical management
Safety guarantee materials on construction site		0.659				Material and technical management
Civilized construction		0.640				Material and technical management
Technical disclosure		0.636				Material and technical management
Emergency drills		0.604				Material and technical management
Experience and skills of workers		0.603				Personnel Management
Workers receive safety training		0.598				Personnel Management
Dangerous material management		0.528				Material and technical management
Workers’ safety protection equipment and living conditions guarantee		0.474				Material and technical management
Efficiency of Security Information Transmission	Information Management		0.944			Information Management
Identification and analysis of risks			0.927			Information Management
Hazard source monitoring			0.782			Information Management
Sharing and learning of safety knowledge			0.748			Information Management
Accuracy of safety information transmission			0.744			Information Management
Investment in safety production expenses			0.435			Material and technical management
Emergency response capability of managers	Personnel Management			0.899		Personnel Management
Safety accomplishment of managers				0.844		Personnel Management
Accident emergency response system				0.694		Organization Management
Organizational competencies of managers				0.663		Personnel Management
Post-disaster recovery system				0.635		Organization Management
Workers’ safety attitude				0.517		Personnel Management
Rationality of organizational model	Organization Management				0.764	Organization Management
Completeness of organizational structure					0.742	Organization Management
Safety supervision system					0.505	Organization Management
Risk management system					0.367	Organization Management

**Table 6:** Results of exploratory factor analysis (EFA) of influence factors of construction safety management.

Dimension		EFA Factor		Related Types of Resilience
T1	Organization Administration	T11	Rationality of Organizational Model	Defense resilience, recovery resilience
		T12	Completeness of organizational structure	Defense resilience, recovery resilience
		T13	Safety supervision system	Defense resilience
		T14	Risk management system	Defense resilience
T2	Personnel Administration	T21	Safety Accomplishment of Managers	Defense resilience, recovery resilience
		T22	Organizational competencies of managers	Defense resilience, recovery resilience
		T23	Emergency response capability of managers	Recovery resilience
		T24	Workers’ Safety Attitude	Defense resilience
T3	Material Technology Management	T31	Risk Control	Defense resilience
		T32	Handling of security incidents	Recovery resilience
		T33	Emergency equipment management	Recovery resilience
		T34	Construction site safety guarantee substance	Defense resilience
		T35	Safe and civilized construction	Defense resilience
		T36	Technical know-how	Defense resilience
		T37	Emergency drills	Recovery resilience
T4	Information Management	T41	Hazard Source Monitoring	Defense resilience
		T42	Identification and analysis of risks	Defense resilience
		T43	Sharing and learning of safety knowledge	Defense resilience, recovery resilience
		T44	Efficiency of safety information transmission	Defense resilience, recovery resilience
		T45	Accuracy of safety information transfer	Defense resilience, recovery resilience

**Table 7:** Fit index calculation.

Index Name	Results	Evaluation
CMIN/df	1.732	Well
RMSEA	0.073	Well
CFI	0.929	Well
TLI	0.920	Well

Note: CMIN is the likelihood-ratio chi square, df is the degree of freedom, RMSEA is the root mean square error of approximation, CFI is the comparative fit index, TLI is the Tacker-Lewis index.

**Table 8:** Regression weights.

Path	Estimate	S.E. (Standard Error)	C.R. (Critical Ratio)	*p*
Organization Management<—The resilience of construction project safety management	0.76	0.620	2.340	0.019
Personnel Management<—The resilience of construction project safety management	0.74	0.558	2.197	0.028
Material Technology Administration<—The resilience of construction project safety management	0.83	0.913	2.563	0.010
Information Management<---The resilience of construction project safety management	0.91			
T14<—Organization Management	0.87			
T13<—Organization Management	0.85	0.068	13.722	***
T12<—Organization Management	0.86	0.064	13.747	***
T11<—Organization Management	0.71	0.077	10.060	***
T24<—Personnel Management	0.82			
T23<—Personnel Management	0.82	0.066	12.396	***
T22<—Personnel Management	0.88	0.078	14.036	***
T21<—Personnel Management	0.84	0.075	13.039	***
T37<—Material Technology Administration	0.87			
T36<—Material Technology Administration	0.83	0.060	12.972	***
T35<—Material Technology Administration	0.86	0.070	14.018	***
T34<—Material Technology Administration	0.79	0.052	12.049	***
T33<—Material Technology Administration	0.77	0.056	11.534	***
T32<—Material Technology Administration	0.89	0.061	15.151	***
T31<—Material Technology Administration	0.83	0.066	13.184	***
T45<—Information Management	0.86			
T44<—Information Management	0.90	0.055	16.278	***
T43<—Information Management	0.81	0.063	12.986	***
T42<—Information Management	0.87	0.053	15.226	***
T41<—Information Management	0.81	0.056	12.977	***

Note: *** *p* < 0.001.

**Table 9:** Key safety management factors in each stage of accident development.

Dimension	Affecting Defense Resilience (before Event)	Affecting Recovery Resilience (When/after Event)
Information Administration	Improve the Effectiveness and Efficiency of Risk Identification and Analysis	Improve the Effectiveness and Efficiency of Safety Information Transmission
	Improving the Effectiveness and Efficiency of Safety Information Transmission	Strengthen the Accuracy of Safety Information Transmission
	Strengthen the Accuracy of Safety Information Transmission	/
Material and Technical Management	Improve the Efficiency of Risk Control	Improve the Efficiency of Handling Safety Accidents
	Promoting Safe and Civilized Construction	Strengthen emergency drills
Organization Administration	Formulate a sound risk management system	Establish a complete organizational structure
	Formulate safety supervision system	/
	Establish a complete organizational structure	/
Personnel Administration	Improve the organizational ability of managers	Improve the organizational ability of managers
	Raise workers’ safety attitude	Improve the emergency response capability of managers
